# Applications of deep convolutional neural networks to predict length, circumference, and weight from mostly dewatered images of fish

**DOI:** 10.1002/ece3.6618

**Published:** 2020-08-04

**Authors:** Nicholas Bravata, Dylan Kelly, Jesse Eickholt, Janine Bryan, Scott Miehls, Dan Zielinski

**Affiliations:** ^1^ Department of Computer Science Central Michigan University Mount Pleasant MI USA; ^2^ Whooshh Innovations, Inc. Seattle WA USA; ^3^ U.S. Geological Survey Millersburg MI USA; ^4^ Great Lakes Fishery Commission Ann Arbor MI USA

## Abstract

Simple biometric data of fish aid fishery management tasks such as monitoring the structure of fish populations and regulating recreational harvest. While these data are foundational to fishery research and management, the collection of length and weight data through physical handling of the fish is challenging as it is time consuming for personnel and can be stressful for the fish. Recent advances in imaging technology and machine learning now offer alternatives for capturing biometric data. To investigate the potential of deep convolutional neural networks to predict biometric data, several regressors were trained and evaluated on data stemming from the FishL™ Recognition System and manual measurements of length, girth, and weight. The dataset consisted of 694 fish from 22 different species common to Laurentian Great Lakes. Even with such a diverse dataset and variety of presentations by the fish, the regressors proved to be robust and achieved competitive mean percent errors in the range of 5.5 to 7.6% for length and girth on an evaluation dataset. Potential applications of this work could increase the efficiency and accuracy of routine survey work by fishery professionals and provide a means for longer‐term automated collection of fish biometric data.

## Graphical abstract

Images of fish used for evaluation. The images are of mostly dewatered fish captured with the FishL™ Recognition system. Length, weight, and girth were predicted from these images.

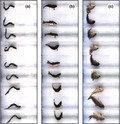

## 1 INTRODUCTION

### 1.1 Value of biometric data

Collection of length and weight data is fundamental to fishery research and management. These metrics form the foundation of fish sample datasets which allow managers to monitor fish populations and identify problems as they arise (Anderson & Neuman, 1996) and provide important insights into fish ecology (Froese, 2006). Length–frequency distributions are commonly used in fishery management to evaluate health of a fish population and identify problems such as overharvest (Anderson & Neuman, 1996; Weithman, Anderson, & Unit, 1978). Managers often monitor the relative condition of fish populations as an indicator of environmental or food supply problems (i.e., diminished prey base). Healthier fish in environments that are supported with sufficient food supplies will have higher relative conditions factors in comparison to unhealthy or starving fish (Anderson & Neuman, 1996; Gabelhouse, 1984). Using simple biometric data, managers can determine a fish population's structure (i.e., the number in each age or size group) and thus the potential for commercial and recreational opportunities. Harvest regulations are often based on fish length so knowing a population's size structure allows managers to track changes in response to harvest regulation (Anderson & Neuman, 1996). Weight data enable calculation of production potential (e.g., kg per hectare) for natural systems (Schaefer, 1965) and if used together with length data can provide estimates of fish health (Bolger & Connolly, Feb., 1989).

### 1.2 Existing approaches to obtain biometric data of fish

Conventional length and weight data collection requires physical handling of fish which is time consuming for personnel and stressful for the fish. Measurements are commonly taken in the field where conditions can be suboptimal for ensuring precision and accuracy. Something as simple as wind, fish bouncing, or differences in measuring techniques among personnel can impact the accuracy of measurements and introduce variability (Gutreuter & Krzoska, 1994). In addition, variability of the fish may introduce error when regression formulas are used to calculate data post hoc. For example, when weight data are unavailable from the field, species‐specific length–weight regression formulas are used to generate weight data (Gerow, Anderson‐ Sprecher, & Hubert, 2005; Murphy, Brown, & Springer, 1990). Unfortunately, morphological variability both among fish and seasonally for individual fish can further reduce the precision of calculated weight estimates (Adams, Leaf, Wu, & Hernandez, 2018; Neumann & Murphy, 1992; Ranney, 2018). Finally, the time and effort required to obtain length and weight measurements in the field imposes limitations on the number of fish that can be sampled; which reduces the confidence in data capturing individual variability (Gutreuter & Krzoska, 1994). For these reasons, a tool or method to increase sampling capacity that allows for measurements on more individual fishes that is standardized, reduces variability in measurements, and captures additional information to calculate weight beyond a simple length–weight relationships would benefit both fish managers and researchers alike.

Various approaches have been tested to automate estimation of fish length and weight, and their use in marine science is likely to increase (MaldeApr. , Handegard, Eikvil, & Salberg, 2019). Ibrahim and Sultana describe image processing such as skeletonization, boundary detection, and machine learning techniques (e.g., Support Vector Machine (SVM), fuzzy classification; Ibrahim & Sultana, 2006). White *et al*. employed image processing algorithms to determine the orientation of a fish and classify as a flatfish or roundfish (White, Svellingen, & Strachan, 2006). The average percent error for these methods generally ranged from 2% to 5%, although many of the methods were species‐specific or involved some manual intervention. Approaches combining optical systems with machine learning report similar error ranges for species‐specific length and weight estimates (Saberioon, Gholizadeh, Cisar, Pautsina, & Urban, 2017). More recently, regional convolutional neural networks (R‐CNN) were used to bound European sea bass in images from a variety of settings, allowing length to be calculated from known‐size fiducial markers (Monkman, Hyder, Kaiser, & Vidal, 2019). Masked R‐CNNs successfully located heads of European hake in images from which head size and thus subsequent overall fish length was estimated (Álvarez‐Ellacuría, Palmer, Catalán, & Lisani, 2020). Finally, large‐segmentation CNNs were able to successfully predict the weight of harvested Asian sea bass and barramundi (Konovalov, Saleh, Efremova, & Domingos, 2019). The use of CNNs for fish biometric data collection is relatively novel yet with technology advancements can be accomplished with little expense (i.e., using commonly available hardware) and under variable conditions (e.g., camera, setting).

Presented here is a deep machine learning approach to predict the length, girth, and weight for multiple species of fish from low‐resolution, dewatered images. Specifically, the regressors were trained and evaluated on a dataset of images for 22 different species common in the Laurentian Great Lakes collected from live fish moving past an image capture device. The goal for this proof‐of‐concept project was to determine whether a deep convolutional neural network (DCNN) could calculate biometric data (length, weight, girth) from fish with limited handling and without a species‐specific classifier.

## 2 METHODS

### 2.1 Data collection

**Figure 1:**
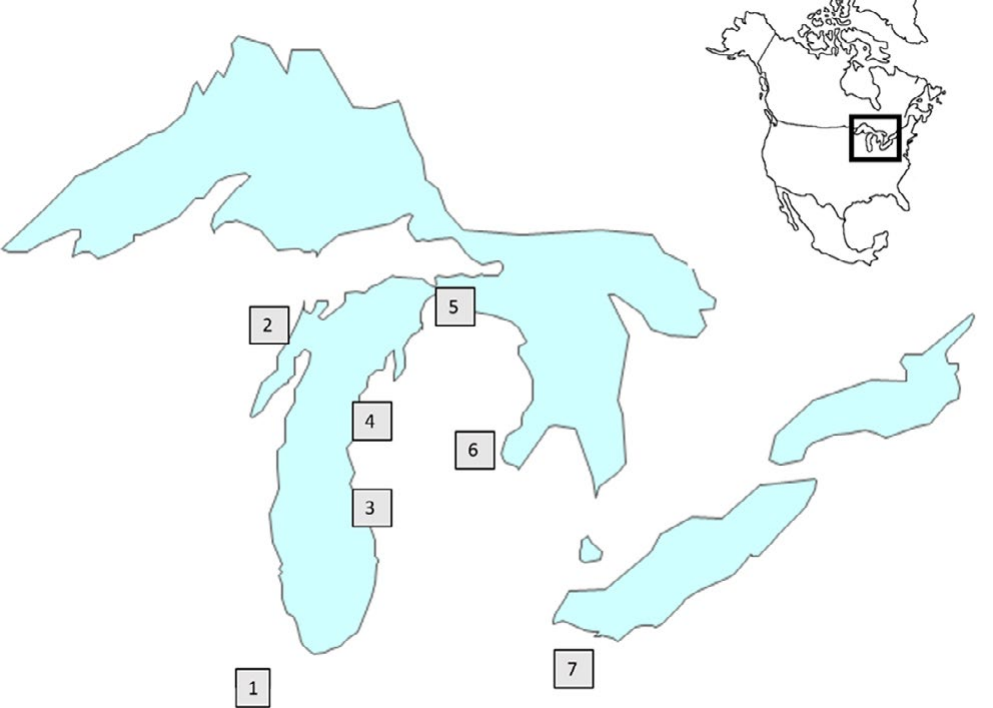
River locations of collected and evaluated fish: (1) Illinois, (2) Menominee, (3) Muskegon, (4) Little Manistee, (5) Cheboygan, Black Mallard, and Ocqueoc, (6) Tittabawassee, and (7) Sandusky

Images and biometric measurements (length, width, girth) were collected for 694 individual fish (22 species) from 9 tributaries (Tittabawassee, Muskegon, Little Manistee, Illinois, Sandusky, Black Mallard, Menominee, Cheboygan, and Ocqueoc Rivers) to the Great Lakes and Mississippi River during spring 2019 (Figure 1). Fish were collected by local state agencies as part of routine assessment and fishery management operations either via electrofishing or netting. Fish were held in livecars instream until processed and then immediately released after images were collected. In the case of silver and bighead carp, the fish were collected as part of an invasive species capture and removal effort and were dead during image collection and measurement. Sea lamprey were collected as part of the Sea Lamprey Control Program assessment operations in tributaries to northern Lake Huron and housed at Hammond Bay Biological Station for other research projects. Fish were identified to species when possible, total length measured to the nearest mm using a 1 m measuring board and weighed to the nearest gram using an electronic scale (MyWeigh model KD‐8000 max weight 8 kg; precision 1 g) or spring scale for fish greater than 8 kg. Girth was measured by wrapping a segment of static net twine around the deepest point of the body, marking where the twine overlapped, and then measuring the marked twine to the nearest mm using the measuring board. After measurements were taken, fish were then passed once through the FishL™ Recognition System (https://www.whooshh.com/scanning‐sorting#OurComponents‐Scanning). Fish were introduced by hand, headfirst, into the imaging system and allowed to slide through on a stream of water as images were automatically captured in less than 0.5 s. The imaging system consisted of an illuminated ramp with six overhead cameras positioned at a fixed distance from the slide. Three cameras captured near‐infrared (IR) images, and three captured color images. Two cameras (one IR and one color) were positioned at three fixed‐angle locations (directly overhead, 45° to left, 45° to right). All images were stored in the portable network graphics (PNG) file format. All recorded biometric data were digitized and then validated against the images by two independent recorders. Table 1 describes key terms and variables used in this manuscript.

**Table 1:** Description of terms used in this article

Name	Comment
10‐fold cross‐validation	With 10‐fold cross‐validation, the dataset is randomly split into 10 equal subsets. One subset is held out as the validation data, and a model is trained on the other 9 subsets. This procedure is repeated 10 times to evaluate each fold, and the results of each iteration are combined as the final estimate of performance.
composite image	The image produced by the FishL^TM^ Recognition System. The composite image includes 18 images taken from 6 different cameras as a fish passes through the system. The composite image contains 9 images from color capture cameras and 9 images from near‐infrared (IR) cameras.
ensemble prediction	A prediction for length, girth, or weight of a fish that was obtained by individually passing the 9 color images of a composite image into a regressor and then averaging the output of the regressor for each image.
multi‐target regressor	A regressor that simultaneously predicts the length, girth, and weight of a fish from a single image.
single image	One of the 9 images in a composite image that was taken with one of the color capture cameras.
single‐model prediction	A prediction that is made using only one single image.
single‐target regressor	A regressor that only predicts one of length, girth, or weight.

### 2.2 Data preparation

**Figure 2:**
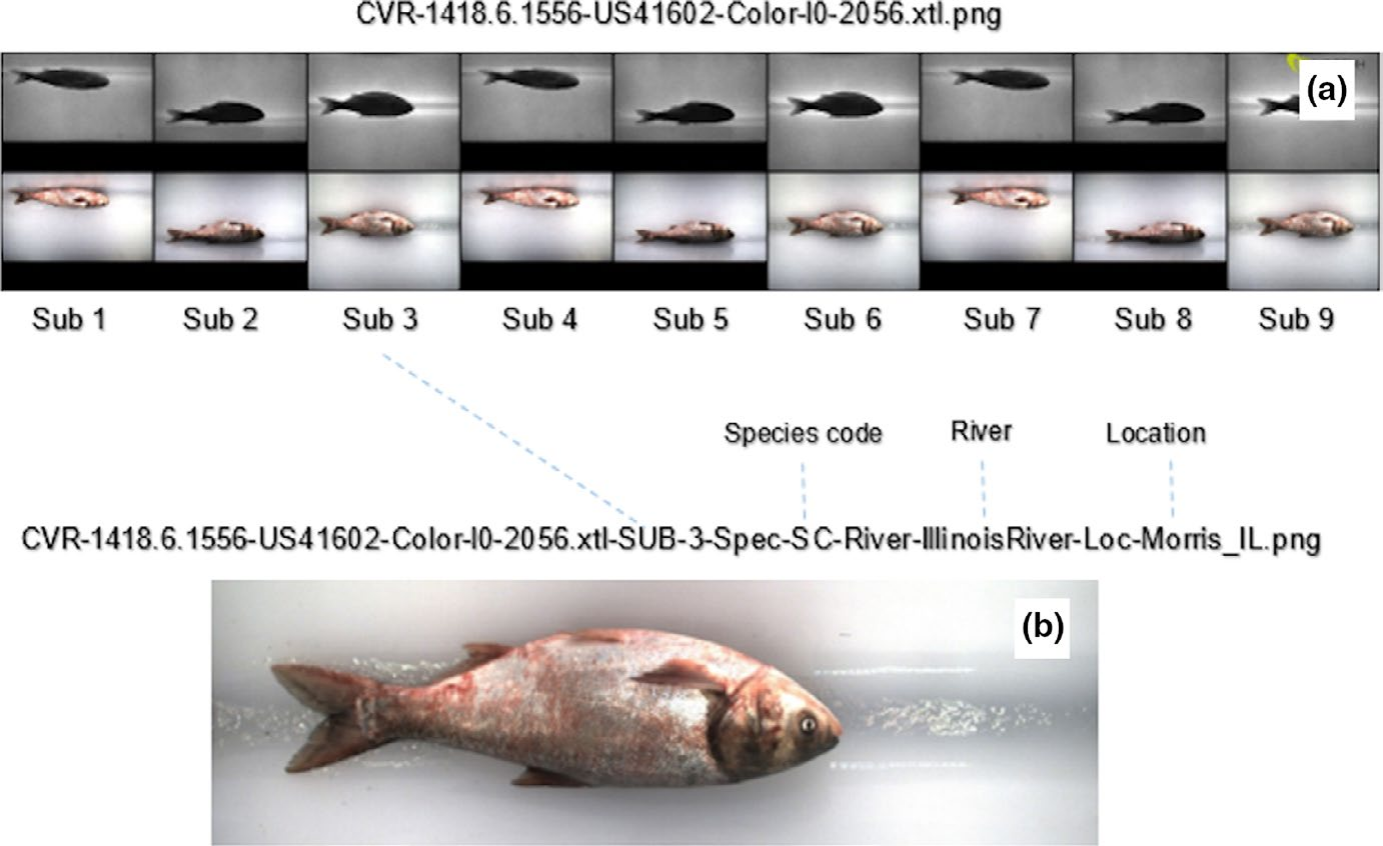
FishL™ Recognition composite (a) of 18 images of a silver carp, and (b) Sub 3, the extracted and expanded third color image

Three pictures were taken in sequence by each of the six cameras as the fish slid through the system generating a composite image file linking the 18 high‐resolution images (see Figure 2).

**Figure 3:**
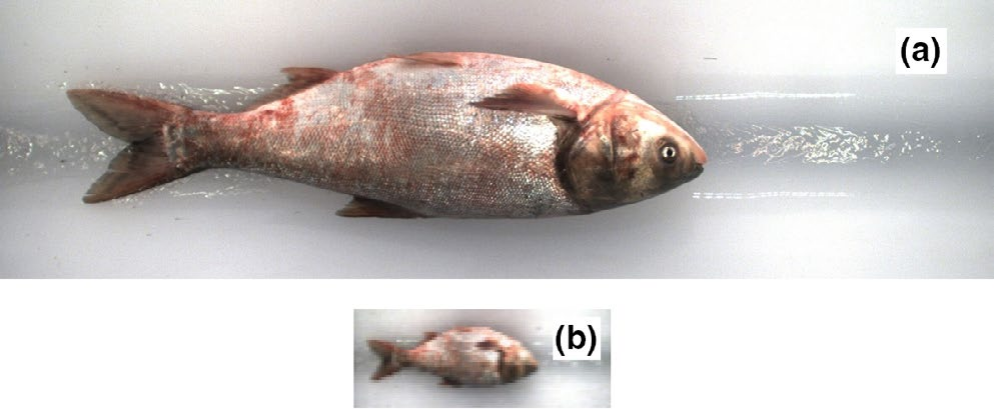
Comparative representation of the original silver carp image from the composite (a) and the extracted and rescaled image (b) image for a silver carp

To increase the number of usable fish images for training and testing, each composite image was broken into single‐individual images. This was done with a custom shell script and the ImageMagick suite (The ImageMagick Development Team, 2020). As each image was extracted, it was rescaled to 75 pixels by 200 pixels, thereby reducing the size of the input into the regressors. Figure 3 is a comparative representation of the image retained after extraction and rescale (B) relative to the original high‐definition color image from the composite (A).

**Figure 4:**
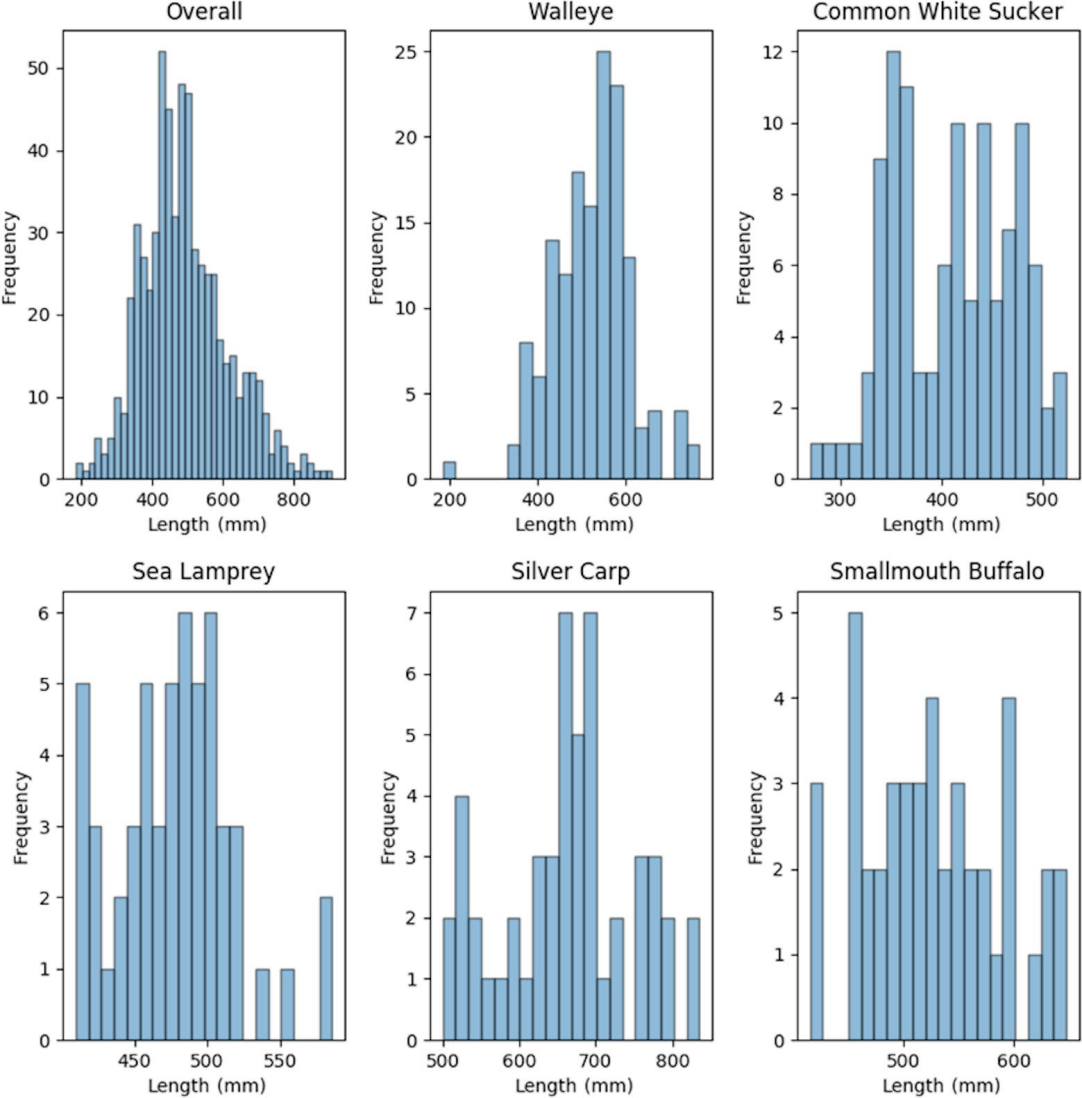
Histograms: Frequency of distribution of fish lengths in the overall training dataset and the top five most abundant species by image count

**Figure 5:**
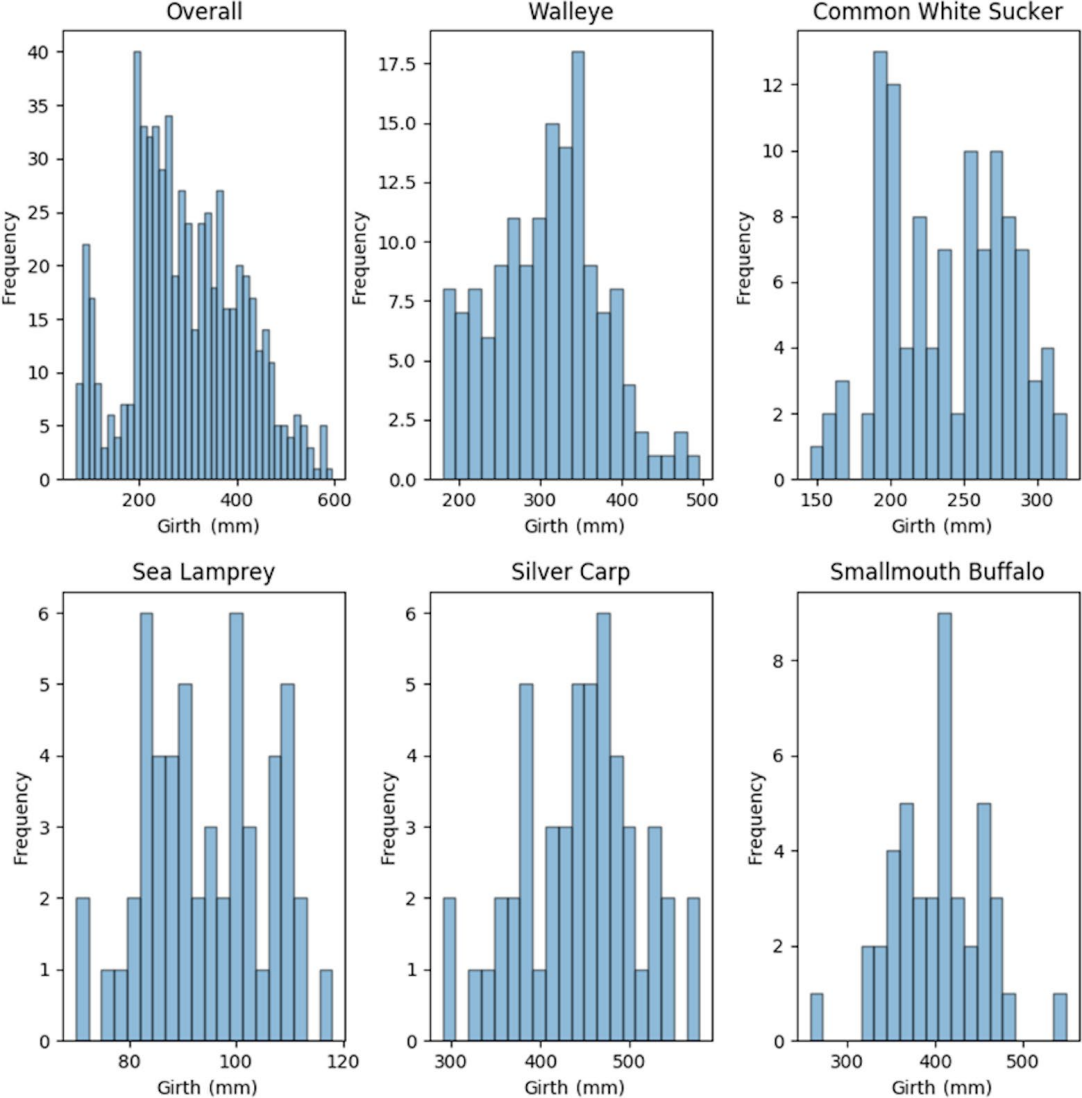
Histograms: Frequency of distribution of fish girths in the overall training dataset and the top five most abundant species by image count

**Figure 6:**
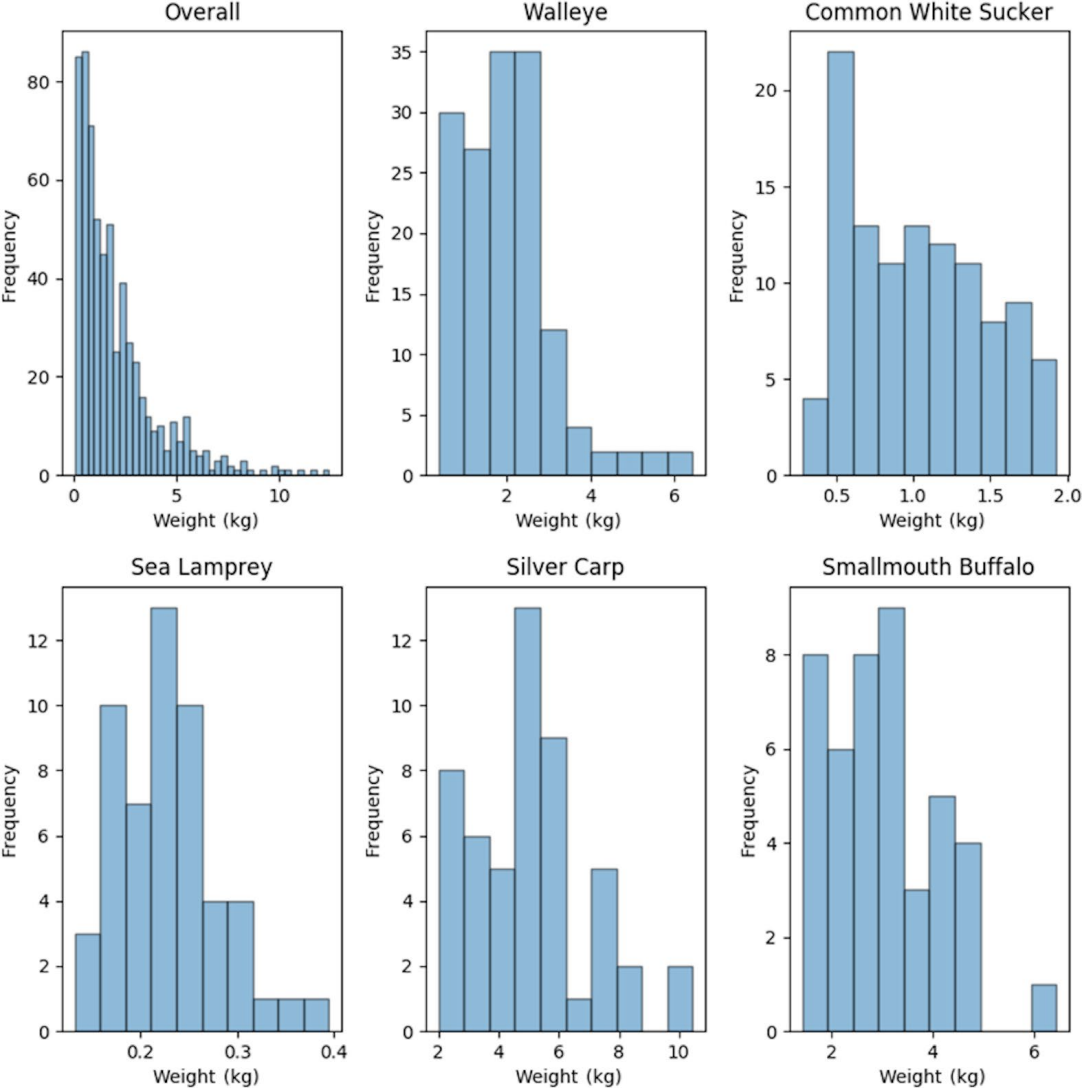
Histograms: Frequency of distribution of fish weights in the overall training dataset and the top five most abundant species by image count

Individual images were extracted from the composites resulting in 6,246 individual images, of which 639 (10%) were randomly selected as the test images. The remaining 5,607 images (90%) were used as training and validation images (Table 2). These training and validation images were segmented into 10‐folds to support a 10‐fold cross‐validation training procedure (def. Table 1). When splitting the data between datasets (and folds), care was taken to ensure that all images stemming from the same composite image were placed in the same dataset (or fold). The test dataset was not used for evaluation until the models had been finalized. Figures 4, 5, 6 show the frequency distribution of lengths, girths, and weights, respectively, of all the fish in the training dataset along with the distributions of the five most commonly represented species.

**Table 2:** Size and composition of validation and test datasets

Species	Number of composite images in the Validation Data	Number of composite images in Test Data
Walleye (WYE)	151	20
Common White Sucker (CWS)	109	7
Sea Lamprey (SL)	54	6
Silver Carp (SC)	51	6
Smallmouth Buffalo (BUF)	44	5
Common Carp (CC)	35	5
Quillback Sucker (QBS)	35	5
Longnose Sucker (LNS)	33	3
Redhorse Sucker (RHS)	28	5
While Bass (WB)	21	1
Bighead Carp (BHC)	18	5
Longnose Gar (LNG)	16	1
White Pearch (WP)	5	0
Smallmouth Bass (SMB)	5	0
Grass Carp (GC)	4	0
Channel Catfish (CCF)	4	0
Northern Hogsucker (NHS)	2	0
River Redhorse Sucker (RRS)	2	0
Goldfish (GF)	2	0
Gizzard Shad (GS)	2	2
Freshwater Drum (FWD)	1	0
Northern Pike (NP)	1	0
Total	623	71

### 2.3 Regressor construction

**Figure 7:**
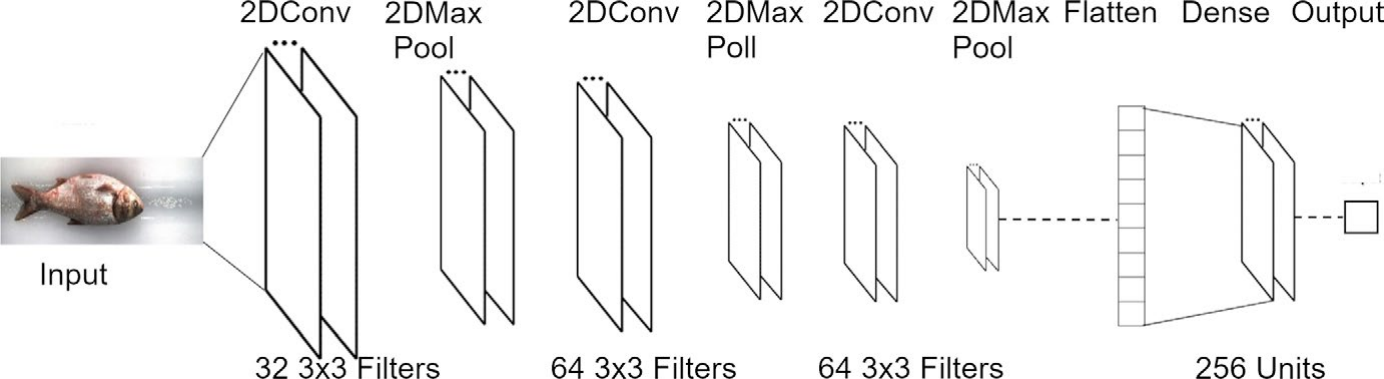
Schematic of the regressor architecture

To predict the length, girth, and weight of a fish, several regressors based on DCNN (Dahl & Sainath, 2013; LeCun, Bengio, & Hinton, 2015; LeCun, Bottou, Bengio, & Haffner, 1998) were constructed. Specifically, they were comprised of three sets of 2D convolutional layers followed by a 2D max pooling layer. The filter size for the convolutional layers was 3 by 3, and those of the max pool layers were 2 by 2. The number of filters was 32 for the first convolutional layer and then 64 for the subsequent layers. The output of the convolutions was passed through one layer of 256 nodes that made use of a rectified linear unit (relu) (Dahl et al., 2013) as an activation function, and the final output of the network was a real value (Figure 7). The input into the regressor was an individual 75 pixels by 200 pixels three‐channel image (see Figure 2b). While several DCNN architectures and training procedures were evaluated, only details and results of the selected architectures and training procedures are reported here. The selections were based on the performance of the regressor to accurately predict the length, girth, and weight of a fish as well as the overall complexity of the DCNN, opting for a simpler model when possible.

### 2.4 Training procedures

Each single‐target regressor (i.e., length, girth, weight) was trained using the same procedure. The input was a single image, and the target was the length, girth, or weight of the fish contained in the image. Training took place over 125 epochs with a batch size of 32. The Adam optimizer (Kingma & Ba, 2014) for Keras (Chollet, 2015) was used to minimize the mean squared error of the target measurement. To increase the effective size of the training data, an image augmentation process was applied (Chollet, 2017) and each training image was randomly rotated (0–15 degrees) or shifted vertically or horizontally (0%–20%). A horizontal flip of the image was also applied on a random basis. No augmentation was performed on the validation nor test images. Additionally, a multi‐target regressor was trained and evaluated. The multi‐target regressor simultaneously predicted the length, girth, and weight of a fish. The Adam optimizer (Kingma & Ba, 2014) for Keras (Chollet, 2015) was used to minimize the average of the mean squared errors across the three measurements. Training of the multi‐target regressor made use of the aforementioned image augmentation process and was done over 125 epochs with a batch size of 32. This regressor was developed as it has been shown that multi‐target regressors tend to produce more robust and generalizable models (Collobert & Weston, 2008; Deng & Yu, 2012; Girshick, 2015; Ruder, 2017).

All regressors were implemented using Keras version 2.1.4 (Chollet, 2015) with TensorFlow version 1.15.2 (Abadi et al., 2016) as the backend. Training and evaluation took place on a Linux machine with twin GeForce GTX 1080Ti graphical processing units. Full details about the runtime environment along with scripts to configure a containerized runtime environment are provided through the links in the Data Accessibility Statement.

### 2.5 Ensemble predictions

To leverage the full extent of the data available for each fish, an ensemble prediction was made from the nine color images of the composite taken of each fish as it passed through the scanning device (see Figure 2 A, second row). To form the ensemble prediction, each of the nine images was passed through a regressor and the output was averaged and taken as the final prediction. For the multi‐target regressor, the averages were taken over the respective targets (i.e., length, girth, and weight).

### 2.6 Evaluation metrics

Mean absolute error (MAE), mean bias error (MBE), and mean percent absolute error (MPAE) were the three metrics used to evaluate the performance of the regressors. The mean absolute error is defined as the mean of the absolute error between predictions and their respective ground‐truth values. More specifically, MAE = $\sum_{i=1}^{n}\left|f\left(x_{i}\right)‐y_{i}\right|/n$ for a dataset with *n* images. *f(x_i_)* is the predicted value for the *i*th image and *y_i_* is the true value. The MBE = $\left(\frac{1}{n}\right)\sum_{i=1}^{n}\left(f\left(x_{i}\right)‐y_{i}\right)$. The MPAE = $100*\left(\frac{1}{n}\right)\sum_{i=1}^{n}\left|f\left(x_{i}\right)‐y_{i}\right|/y_{i}$. Given the variation of the ground‐truth values, the MPAE provides a more robust measure of performance across metrics (e.g., an absolute error of 0.5 cm would represent a percent error of 10% for a fish measuring 5 cm in length but that same absolute error would only represent a percent error of 2% for a fish measuring 25 cm in length).

## 3 RESULTS

The first set of models evaluated were single‐target regressors that predicted the length, girth, or weight of a fish. On the test data, the ensemble predictions from the single‐target regressors performed comparably to the single‐model predictions across all three types of measurements in terms of MPAE (i.e., 8.3%–7.6% for length, 17.3%–16.8% for girth, and 28.6%–26.9% for weight; Table 3).

**Table 3:** Performance of single‐target regressors on validation and test datasets

Regression tasks (model type)	Validation Dataset (*n* = 623)			Test Dataset (*n* = 71)		
	MAE	MPAE	MBE	MAE	MPAE	MBE
Length (single target; single model)	30.5 mm	6.7%	0.54 mm	40.1 mm	8.3%	30.9 mm
Length (single target; ensemble)	25.4 mm	5.6%	0.54 mm	36.6 mm	7.6%	30.9 mm
Girth (single target; single model)	23.8 mm	9.1%	−3.47 mm	43.0 mm	17.3%	36.6 mm
Girth (single target; ensemble)	20.3 mm	7.7%	−3.47 mm	41.3 mm	16.8%	36.6 mm
Weight (single target; single model)	0.324 kg	24.3%	0.0 kg	0.661 kg	28.6%	0.2 kg
Weight (single target; ensemble)	0.261kg	19%	0.0 kg	0.634 kg	26.9%	0.2 kg

**Figure 8:**
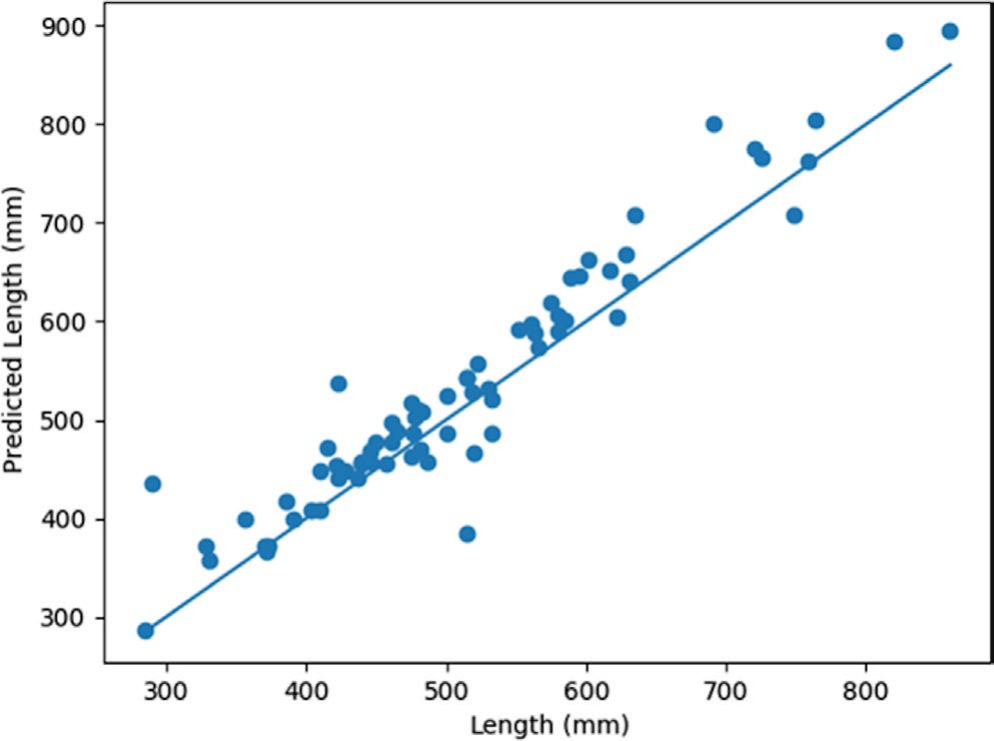
Multi‐target regressor ensemble predictions for length versus measured length on the test dataset

**Figure 9:**
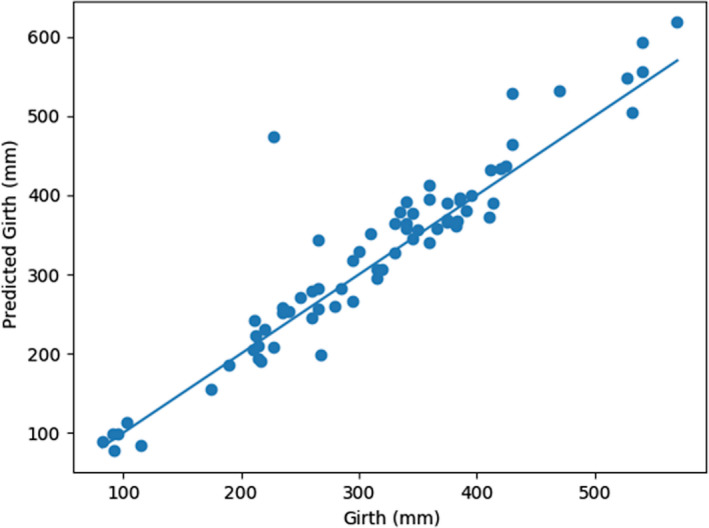
Multi‐target regressor ensemble predictions for girth versus measured girth on the test dataset

**Figure 10:**
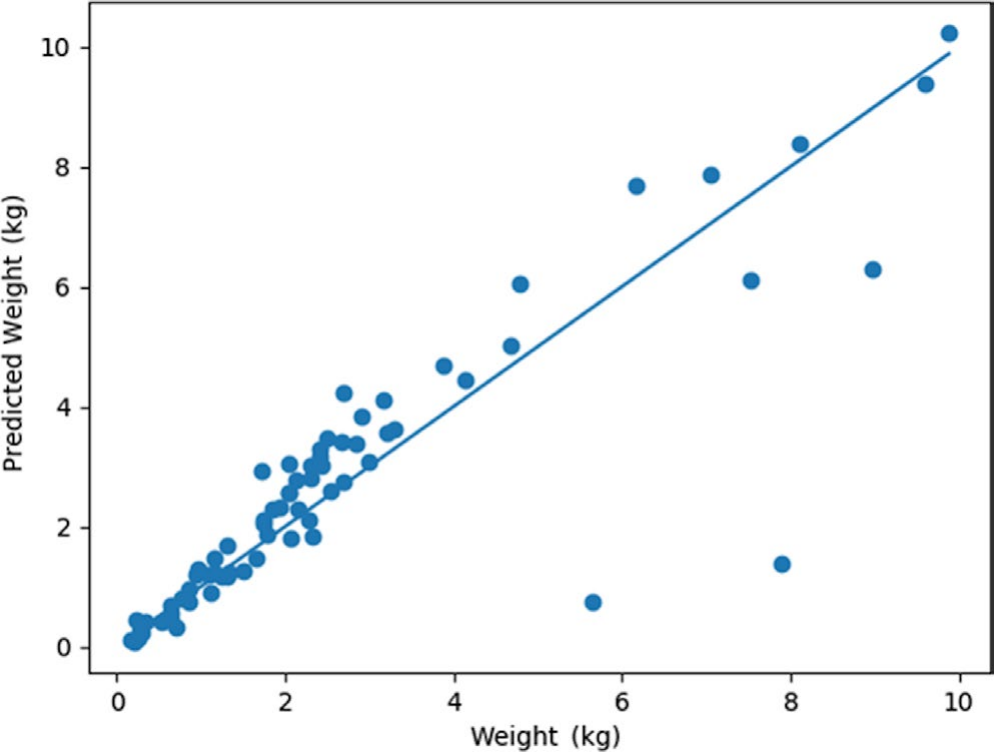
Multi‐target regressor ensemble predictions for weight versus measured weight on the test dataset

**Figure 11:**
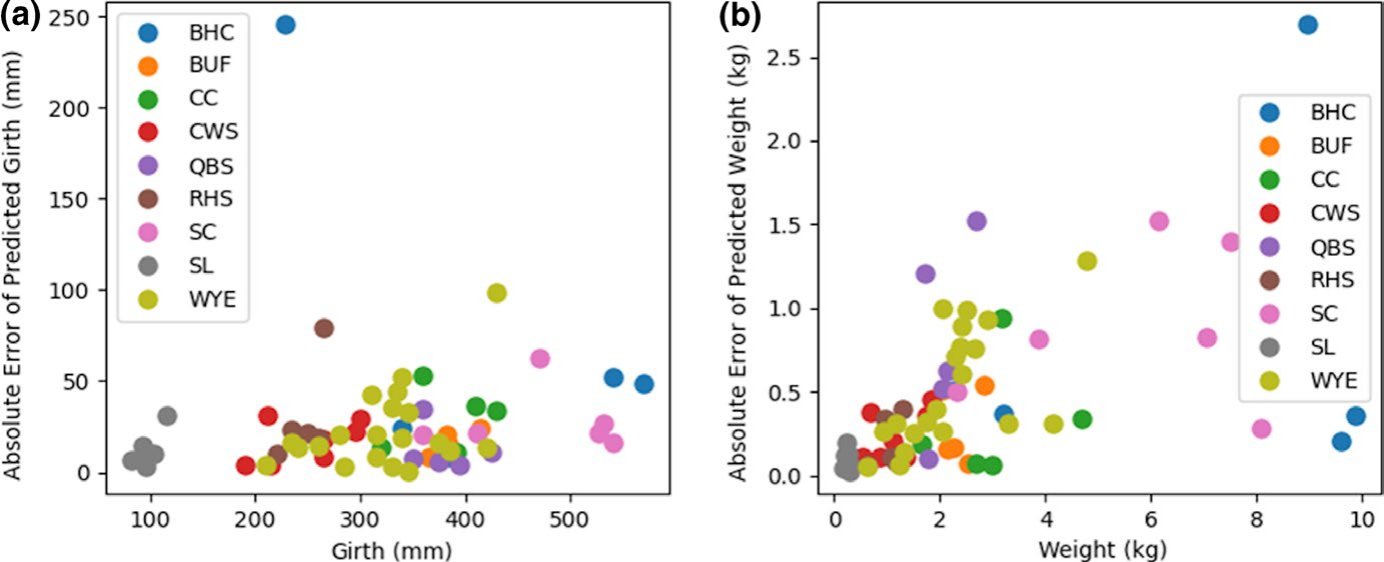
Absolute error of multi‐target ensemble predictions for girth (a) and predicted weight (b) on the test dataset. The predictions for species with 5 or more samples in the test dataset are plotted against the measured girth and weight, respectively

**Figure 12:**
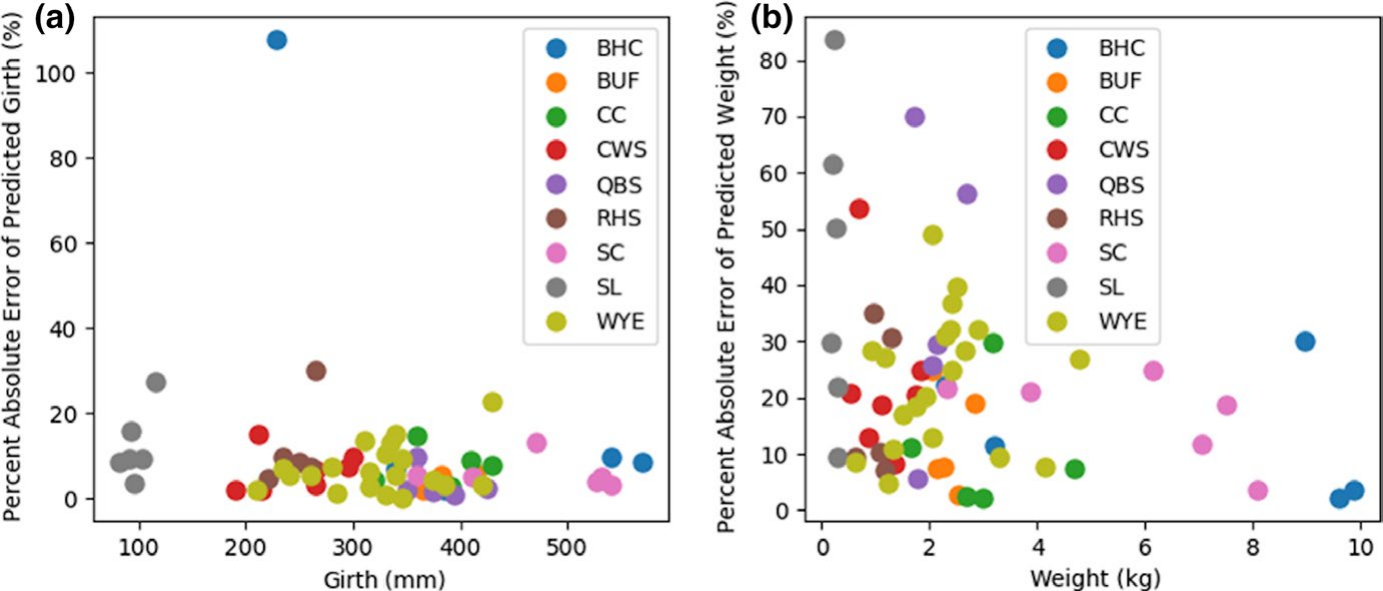
Absolute percent error of multi‐target ensemble predictions for girth (a) and predicted weight (b) on the test dataset. The predictions for species with 5 or more samples in the test dataset are plotted against the measured girth and weight, respectively

The second set of models evaluated were multi‐target regressors. These models were trained to predict the length, girth, and weight of a fish (i.e., for one input three separate values were predicted). When comparing the results of the multi‐target regressors to the single‐target regressors on the test dataset, the MBE is less for the multi‐target regressors than single‐target regressors across all three measurement types (i.e., 30.9 mm to 21.6 mm for length, 36.6 mm to 11.53 mm for girth, and 0.2 kg to 0.1 kg for weight; Table 4). The MPAE and MAE are also less for the multi‐target regressors than the single‐target regressors across the length and girth outcomes of the test dataset. The trend lines drawn through the plotted multi‐target regressor data points relative to actual measured values visibly highlight the differences in the regressor predictions outcomes with a range of values wherein the predictions were tight and a range across which regressor biases were evident (Figures 8, 9, 10).The regressor tends to overpredict length for longer fish and underpredict weight for heavier fish. Comparing the girth and weight MAE and MPAE versus actual measured values shows a much tighter clustering of the data points in the girth plots indicating overall reduced error in the multi‐target regressor predictions for girth relative to weight (Figures 11 and 12). Body shape and size of the different fish species did not influence the performance of the multi‐target regressor at predicting girth. However, for weight the MAE of a portion of Quillback Suckers and Silver Bighead Carps was slightly higher. All three of these species share a similar body shape. Sea Lamprey, a light weight tubular‐shaped species exhibited the most significant divergence of MPAE of predicted weight distribution which was replicated to a lesser degree by the Common White Sucker, also a somewhat tubular‐shaped species.

**Table 4:** Performance of multi‐target regressors on validation and test datasets

Regression tasks	Validation Dataset (*n* = 623)			Test Dataset (*n* = 71)		
	MAE	MPAE	MBE	MAE	MPAE	MBE
Length (multi‐target; single model)	31.9 mm	6.8%	8.2 mm	36.3 mm	7.4%	21.6 mm
Length (multi‐ target; ensemble)	27.6 mm	6.0%	8.2 mm	32.2 mm	6.5%	21.6 mm
Girth (multi‐ target; single model)	23.4 mm	9.2%	8.6 mm	30.0 mm	10.9%	11.53 mm
Girth (multi‐target; ensemble)	19.9 mm	7.8%	8.6 mm	26.0 mm	9.2%	11.53 mm
Weight (multi‐ target; single)	0.57 kg	59.8%	0.4 kg	0.68 kg	33%	0.1 kg
Weight (multi‐target; ensemble)	0.5 kg	52.8%	0.4 kg	0.59 kg	24.0%	0.1 kg

## 4 DISCUSSION

It is difficult to draw direct comparisons with the prior work reviewed by Ibrahim & Sultana (2006) and Saberioon et al. (2017) as those approaches were evaluated on at most a few species. Still, the results of our single‐target ensemble regressors for the predicted length and girth on the cross‐validation dataset are comparable to the species‐specific models. The ground‐truth values for length, girth, and weight were taken in the field and likely exhibit some inherent variability.

The diversity of the dataset in terms of species likely added to the difficulty in predicting the weight of the fish from the images. The length of the fish directly correlates to pixels occupied by the fish in the image. For girth and weight, the relationship between pixels occupied and the girth may vary depending on the species of fish (e.g., the side profile of two fish may be similar in size in the image but the weight may differ depending on the common cross‐sectional shape of a species of fish). An additional species‐specific challenge for weight is the fins. The relationship between the surface area of a fish in an image and its weight will depend on the percentage of the surface area that relates to fins (e.g., images of two fish may occupy the same surface area but if the surface area covered by fins is less in one image, then the weight of one fish may be greater than the other). Additional data or providing species information would likely improve a regressors' performance for girth and weight.

### 4.1 Potential use cases for predicted biometrics

An automated approach to collecting length and weight data would allow fisheries professionals more time to devote to catching fish and processing data. An automated tool could be incorporated into routine survey work in which personnel collect and pass fish through an image capture device to either store images for later analysis or even process images in real time for infield prediction of metrics and indices. Image data for this study were collected as part of routine fishery management assessment efforts. By far, the most time‐consuming part of the data collection was measuring and weighting the fish, whereas image capture required considerably less time than the manual data collection. Beyond routine assessment work, image capture and analysis tools could be incorporated into scenarios where fish are concentrated and moving past a fixed location such as fish passage structures (Garavelli et al., 2019). Automated image capture and processing in this context would provide managers an accurate assessment of the species being passed and corresponding sizes and conditions without the need for personnel on‐site, handling every fish for weeks or even months. Real‐time assessment of length and girth at passage scenarios could also be used to sort fish based on size (Garavelli et al., 2019).

### 4.2 Robustness of predictions across presentations

**Figure 13:**
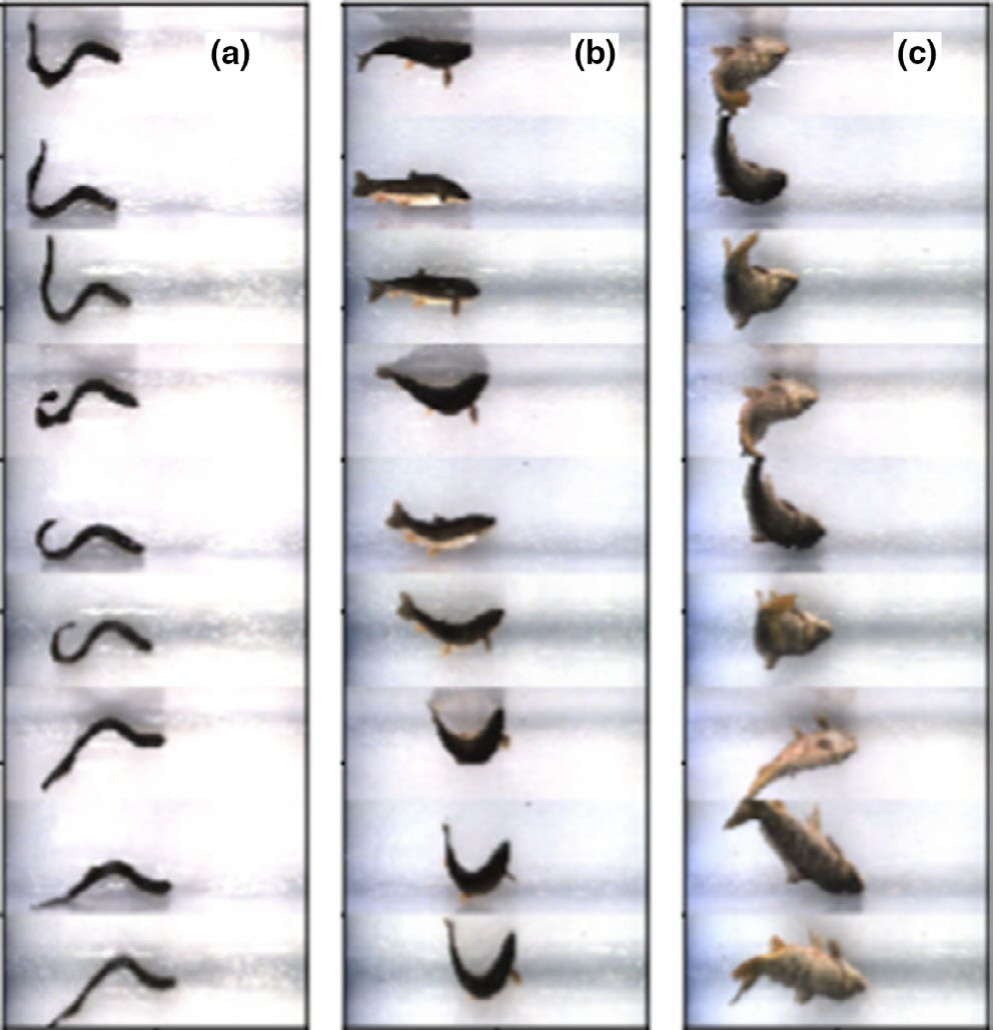
Ensemble images for three fish from the test dataset that were parallel with the plane of the image (Panels a, b) and perpendicular to the image (Panel c). The regressors perform well when fish are parallel with the image and poorly when perpendicular

An advantage of the ensemble prediction approach is that it leverages several images of a fish when making a prediction, reducing the likelihood of a poor prediction due to a poor presentation of the fish. Using an example of three fish from the test dataset (Figure 13) with recorded lengths of 482, 410, and 290 mm, the ensemble approach predicted lengths of 497, 409, and 445 mm. In spite of the extensive movement evidenced in the images, two of the predictions were within the average MAE reported over all the images from the test dataset. Most of the images of the fish in Figure 13 C were taken down the length of the fish as it had jumped when the images were captured. This presentation did not provide a good lateral view of the fish and resulted in a very poor prediction.

### 4.3 Limitations

While these results are promising, they should be interpreted with caution. As the histograms indicate, most of the data used for the construction and evaluation of biometric predictions stem from adult fish and therefore do not constitute the full range of lengths, weights, and girths possible for the included species. A more uniformly distributed dataset across species and age would provide additional support for the generalizability of the regressors. Still, bias to adult fish is not unique to this project and is a function of standard sampling protocol that typically emphasizes adults (i.e., mesh sizes; Pope & Willis, 1996).

The current approach to use all the images for a fish as input to a regressor is limited in that it does not make use of the order in which the images of the fish were captured. At present, each image is processed individually, irrespective of the other eight images or the position of the camera. If all nine images were used as input, there may be information that can be leveraged from the camera angle or the movement between frames (i.e., recall that the nine images are taken as a series of three images and as a result capture some movement patterns). In this particular work, the limited amount of data did not support the development of such multi‐image regressors. Doing so would have reduced the amount of training and evaluation data by a factor of nine.

The generalizability of the regressors is likely limited in settings beyond the capture technology used in this work. The mostly dewatered and lateral presentation of the fish provides a high‐quality input to the DCNN. The uniform background reduces the complexity of the regression task, and multiple images captured by the scanning device reduce the error introduced by poor presentations. In addition to aiding with the quality of the input, the scanning device also ensures a constant distance between the camera and the fish. The models presented were not architected to accommodate variable distances between the camera and the fish and are unlikely to generalize to other distances. Nevertheless, these results demonstrate what is possible with currently available data capture technology.

### 4.4 Additional directions of study

An additional line of investigation could be the effect of species information as an additional input to the regressors. While such information is not always available, there are situations when it is (e.g., during targeted species collection, visual inspection by personnel handling the fish) and using this extra information may provide for more precise predictions. Using the data available in this study, additional regressors were trained that used species information as an additional input (data not reported). The models exhibited a large amount of overfitting (i.e., the models did not generalize well to the data in the test dataset). With additional training data, the value of species information could be further investigated.

## 5 CONCLUSION

Presented here is an overview and evaluation of a set of novel regressors to predict length, girth, and weight of fish from images of mostly dewatered fish. The images stemmed from nine color images of a FishL™ Recognition System, captured as a fish passed through a 1.5 m chute. Single‐image target‐specific regressors for length, girth, and weight achieved a mean percent absolute error of 8.3, 17.3, and 28.6, respectively, on a test dataset. Ensemble target‐specific regressors that utilized all nine images of a fish achieved a mean percent absolute error of 7.6, 16.8, and 26.9, respectively, on the same test dataset. A multi‐target ensemble regressor was able to achieve a mean percent error of 6.5, 9.2, and 24.0. In general, the regressors’ predictions for length are robust with respect to the presentation of the fish as it passes through the image capture device. Potential applications of this work could increase the efficiency and accuracy of routine survey work by fishery professionals and provide a means for longer‐term automated collection of fish biometric data.

## CONFLICT OF INTERESTS

The manuscript describes application of deep learning on images which were captured by a new technology, the FishL™Recognition System, designed and developed by the company at which Janine Byran is employed, Whooshh Innovations, Inc. The use of the system and the subsequently processed images were provided as an in‐kind service by Whooshh to further the scientific inquires of Great Lakes Fishery Commission and Central Michigan University with regard to the future potential of automated prediction of biometric data facilitating fisheries management. Whooshh Innovations, Inc. is a private corporation. All employees hold company stock units and participate in the Employee Stock Option Program. The other authors have no competing interests to declare.

## AUTHOR CONTRIBUTION

**Nicholas Bravata:** Formal analysis (equal); Software (lead); Writing‐original draft (supporting). **Dylan Kelly:** Formal analysis (equal); Software (supporting); Writing‐original draft (equal). **Jesse Eickholt:** Conceptualization (equal); Data curation (equal); Formal analysis (equal); Funding acquisition (equal); Methodology (equal); Software (equal); Visualization (lead); Writing‐original draft (equal); Writing‐review & editing (equal). **Janine Bryan:** Data curation (equal); Funding acquisition (equal); Writing‐original draft (equal); Writing‐review & editing (supporting). **Scott Miehls:** Conceptualization (equal); Formal analysis (equal); Funding acquisition (equal); Supervision (equal); Writing‐original draft (equal); Writing‐review & editing (equal). **Daniel Zielinski:** Conceptualization (equal); Formal analysis (equal); Funding acquisition (equal); Writing‐original draft (equal); Writing‐review & editing (equal).

## Data Availability

The extracted images and measurement data are available on OSF at https://doi.org/10.17605/OSF.IO/KQVG8. The scripts used for training and evaluation and the models used to evaluate the test data are available on OSF at https://doi.org/10.17605/OSF.IO/VKXJM.
